# Nanoparticle tools for maximizing oral drug delivery

**DOI:** 10.1590/1414-431X2025e14459

**Published:** 2025-06-16

**Authors:** D.M. Cahyani, A.S. Mubarok, B.S. Hariawan, I. Amalina, P. Drake, T. Parumasivam, R.K. Sahu, M.A.S. Rijal, R. Sari, A. Miatmoko

**Affiliations:** 1 Master Program of Pharmaceutical Sciences, Faculty of Pharmacy, Universitas Airlangga, Campus C UNAIR Mulyorejo, Surabaya, Indonesia; 2 Nanotechnology Engineering, Faculty of Advanced Technology and Multidiscipline, Universitas Airlangga, Campus C UNAIR Mulyorejo, Surabaya, Indonesia; 3 School of Chemistry and Biosciences, Faculty of Life Sciences, University of Bradford, Bradford, UK; 4 School of Pharmaceutical Sciences, Universiti Sains Malaysia, Pulau Pinang, Malaysia; 5 Department of Pharmaceutical Sciences, Hemvati Nandan Bahuguna Garhwal University (A Central University), Chauras Campus, Tehri Garhwal, Uttarakhand, India; 6 Department of Pharmaceutical Sciences, Faculty of Pharmacy, Universitas Airlangga, Campus C UNAIR Mulyorejo, Surabaya, Indonesia; 7 Pharmaceutics and Delivery Systems for Drugs, Cosmetics, and Nanomedicines Research Group, Faculty of Pharmacy, Universitas Airlangga, Campus C UNAIR Mulyorejo, Surabaya, Indonesia; 8 Stem Cell Research and Development Center, Universitas Airlangga, Institute of Tropical Disease, Campus C UNAIR Mulyorejo, Surabaya, Indonesia

**Keywords:** Human health, Nanoparticle, Oral drug delivery, Solubility, Permeability

## Abstract

The biological permeability and water solubility of drugs can pose substantial obstacles to oral drug delivery, the most common mode of drug administration for improving human health. Solubility determines the amount of drug that can be dissolved in solution, whereas permeability is the ability to permeate across biological membranes, determining therapeutic efficacy and safety. Some biological barriers, such as gastrointestinal pH, enzymes, and mucus, may affect the dissolution or absorption of therapeutic drugs. Physical or chemical approaches can be used to modify the water solubility or enhance the permeability. Moreover, nanocarriers, which can increase drug stability through encapsulation, enhance absorption due to their extensive surface area, and facilitate the targeted administration of medications to certain areas, could be useful for drug delivery systems. Nanoparticles can increase drug solubility by particle size reduction, complexation, and drug encapsulation and increase permeation by retention in tumors, opening of tight junctions, membrane fluidization, or intestinal mucoadhesion. Despite the many advantages of nanoparticle drug formulations, they also have several limitations, such as complicated manufacturing processes, nanotoxicity, and stability issues. In this article, we provide a comprehensive description of nanoparticle tools for maximizing oral drug delivery.

## Introduction

Non-invasive oral, intranasal, and topical drug delivery is more desirable than parenteral drug delivery. For example, oral drug administration prevents toxicity and improves the quality of disease management. Oral drug delivery is preferred over other non-invasive routes because of its proven safety, ease of use, and therapeutic efficiency (1). Based on reports (2), around 60% of commercially available small molecule therapeutic products are taken orally, and oral formulations account for 90% of the global market for all pharmaceutical formulations.

Despite the advantages of oral administration of drugs, several challenges are encountered in oral drug formulations because of the physical characteristics of the medication, such as solubility and permeability. A significant issue that the pharmaceutical industry encounters is the limited ability of active pharmaceutical ingredients to dissolve in water (3). According to Noyes and Whitney equation (4), solubility has the greatest influence on the bioavailability of drugs because of its role in the dissolution process. Solubility is a thermodynamic property that indicates the amount of a substance that may be dissolved in a given solvent at equilibrium. Therefore, solubility becomes an important physicochemical property of active pharmaceutical compounds that needs to be explored and modified (5).

Studies have shown that 40% of new drug compounds and other existing drugs have poor solubility, which hampers oral drug bioavailability (6). The limited solubility of drugs in water is mainly caused by the hydrophobicity of the molecule, which hinders its ability to form hydrogen bonds with water. Additionally, a high crystal lattice energy can impede the breakdown and dissociation of drug molecules, preventing the drug to form a solution (7). Hydrophobic drugs refer to a wide range of compounds with poor solubility in water but that can dissolve in organic solvents. Drug compounds can be categorized according to their solubility in water; drugs that dissolve in less than one part of water are considered very soluble, those requiring between 1-10 parts are considered soluble, and the ones requiring more than 10,000 parts of water are considered practically insoluble.

Orally administered drugs must be absorbed from the gastrointestinal tract before they can be delivered to the intended target areas and produce their therapeutic effects (5). For this reason, oral drug formulations have poor permeability. Drug permeability is a measure of how easily a compound crosses a membrane. In oral drug delivery, permeability is associated with how much a drug can penetrate the intestinal wall per unit of time (8). This parameter determines the speed at which the dissolved drugs traverse the intestinal wall and enter the systemic circulation. Drug absorption is heavily influenced by permeability, which is an intricate dynamic procedure that relies on many physiological and physicochemical features of the gastrointestinal membrane (5). The permeability of pharmacological molecules across the intestinal membrane is crucial in determining their pharmacokinetic profile. The small intestine accounts for approximately 90% of drug absorption (4).

Insufficient bioavailability can be due to various causes, including low drug solubility, slow drug dissolution rate, and inadequate drug permeability in the gastrointestinal system. Furthermore, the absorption of drugs through the oral route is hindered by low drug stability in gastrointestinal tracts and several physiological obstacles, including pH levels, efflux transporters, and metabolic enzymes (2). The Biopharmaceutics Classification System (BCS) of the Food and Drug Administration (FDA) highlights water solubility and drug permeability across the intestinal membrane as the primary parameters influencing oral drug absorption (4). Solubility is a crucial factor in the gastrointestinal tract that affects how much drug molecules can be absorbed into the bloodstream when taken orally (9). In particular, there is a need to enhance the solubility and permeability of BCS class IV medicines, which currently have low levels of both properties. Increased solubility and permeability can also increase the bioavailability of orally administered drugs.

Various methodologies and formulations have been devised to improve solubility, absorption, and/or bioavailability of active pharmaceutical ingredients. Some conventional approaches include micronization, addition of penetration enhancers or cosolvents, creation of surfactant-based dispersion systems, and salt formation. However, these strategies have limitations in enhancing the solubility of active pharmaceutical ingredients with low water solubility. In recent decades, nanotechnology has been utilized to create nano-based systems that help in administering medicines in many medical domains. Nanoparticle drug delivery methods have been used to alter the physicochemical properties, thus enhancing pharmacokinetic and pharmacodynamic profiles of several drugs for therapeutic purposes (2).

This review focused on strategies for utilizing current technologies to increase drug solubility and permeability, addressing the current challenges in oral drug formulation. In addition, we investigated the potential method by which nanoparticles can be used as carriers for medication delivery to enhance drug solubility and permeability, and thus improve oral drug bioavailability.

## Search strategy

Multiple databases, including PubMed and Scopus, were searched to identify the publications discussed in this narrative review. The reviewed manuscripts were published until June 2024, and the most current ones were included.

### Biological barriers to oral drug delivery

The most popular and recommended method of taking medication is orally. However, it can be impacted by issues like gastrointestinal stability, pre-systemic breakdown, and limited permeability. The absorption of oral drugs is a complicated process that depends on a number of gastrointestinal physiological and drug-related parameters. Drugs taken by mouth are digested in the stomach and processed by acids and digestive enzymes. Ultimately, the drug reaches the small intestine where it releases active molecules and continues to be absorbed through simple diffusion, enhanced diffusion, or active transport (4).

The stomach has a highly acidic environment with a pH of 1.0 to 2.5, which may break down acid-labile drugs, making it the most difficult organ for the absorption of pharmaceuticals (10). However, due to the limited surface area (11), drugs have a low absorption in the stomach and rapidly traverse to the small intestine, where the majority of absorption takes place. Drugs that have limited absorption are formulated to be released slowly in the colon (12).

The longest and most intricate portion of the digestive system is the small intestine, measuring around 6 meters in length. The small intestine is also regarded as the primary organ of drug absorption due to its enormous surface area. The existence of villi and microvilli, which are densely packed with blood vessels, allows an even greater surface area. However, the absorption of several drugs is restricted by barriers such as extreme pH, mucus, tight junctions, efflux transporters, and enzymes (4).

### Mucous hydrophilic layer

The gastrointestinal mucus consists of mucin (2-5% w/v), glycans, and trace amounts of proteins and lipids. Food, chyme, and excrement travel more easily through the gastrointestinal system because mucus lubricates the gastrointestinal epithelium. In addition to permitting nutrition exchange, mucus shields the epithelium from pathogens, poisons, and endogenous chemicals like pepsin, hydrochloric acid, and other digestive enzymes (13).

A thick layer of mucus can affect drug absorption. As reported previously, the thickness of the mucosal layer varies according to the pathophysiological state, i.e., gastritis and gastrointestinal ulcers, the interactions with the external environment, and the gastrointestinal tract's physical region. Irritation of the digestive tract stimulates mucosal thickening, reducing the ease with which drugs can pass through the mucosa and reach the epithelium. Consequently, one of the main obstacles to medication absorption is the mucosal layer (4).

### pH of the gastrointestinal tract

Varied sections of the gastrointestinal tract have varied pH values (14). A quantitative meta-analysis was conducted to examine the pH values and variability in different parts of the gastrointestinal system. The authors demonstrated that the caloric content of the food had a substantial impact on both the mean pH and pH variability of the stomach, with an increase in caloric content also leading to an increase in gastric pH variability. Nonetheless, eating has little effect on the pH of the colon and intestines (15).

Alterations in the gastrointestinal tract pH may impact the solubility, release, stability, dissolution, and permeability of the drug (14). The solubility of medications that are weak acids or bases is also impacted by pH. Weakly basic drugs are more soluble in the stomach's acidic environment. As medications become less soluble in an increasingly alkaline environment, precipitation occurs. On the other hand, weakly acidic medications dissolve very little in the stomach and become more soluble as they move through the more alkaline environment of the small intestine (14).

### Enzymes and bile salts

In the gastrointestinal tract, drugs and dosage forms may be impacted by enzymatic and microbiological degradation. The primary gastric and intestinal enzymes metabolize carbohydrates, lipids, and proteins. The gastric and small intestine's primary food-digesting enzymes affect drug stability, which must be considered when designing a formulation for localized drug administration in the gastrointestinal system (11).

The solubility and pace of drug dissolution in the intestine, particularly for hydrophobic medicines, can also be influenced by the composition of bile salts. Due to the digestion, absorption, and release of pancreatic and bile fluids into the intestinal lumen, the total composition of intestinal fluids varies during intestinal transit. Bile salt concentrations during fasting vary from 1.4 to 5.5 mM in the jejunum and from 2.5 to 5.9 mM in the duodenum (12).

### Solubility affects drug bioavailability

Drug solubility in water is a fundamental property in drug absorption after oral administration since it is necessary to get the drug's desired concentration into the systemic circulation. A precise drug concentration elicits the desired physiological response. Because water is the most common solvent for liquid pharmaceutical formulations, any drug to be absorbed must be presented as an aqueous solution at the absorption site. Drugs with poor water solubility are absorbed slowly, resulting in insufficient bioavailability (1). Consequently, larger dosages are needed to attain therapeutic plasma concentrations. In other words, considering the fact that any medicine that is absorbed must be in solution form at the site of absorption, low water solubility is a major issue that arises during the creation of new chemical formulations as well as generics (16).

The BCS is a reference that uses intestinal solubility and permeability factors to predict drug absorption in the gastrointestinal tract. The solubility level of an immediate-release product is determined by its greatest dose strength. When the drug can dissolve at the maximum dosage in 250 mL or less of an aqueous medium with a pH of 1-7.5, it is said to be very soluble. This volume estimate is derived from a standard bioequivalence study, in which a medicinal product and a glass of water are administered to a fasting volunteer (16). Most medications now in use, especially those in the BCS Class II category, are weak bases or acids with low solubility in water. Rather than drug absorption, the release of the drug from the dosage form and its solubility at gastric pH are the rate-limiting steps for medicines with poor solubility (16). Many methods have been developed to improve the solubility of drugs.

### Permeability affects drug bioavailability

The design of the drug formulation as well as a number of biopharmaceutical characteristics pertaining to the anatomy and physiology of the gastrointestinal system influence drug permeability. Another important aspect influencing absorption and distribution is the permeability of cell membranes. Mucus is a barrier to drug absorption that many drugs must pass before being absorbed. Physicochemical characteristics of the medication, such as its particle size, solubility, and lipophilicity, influence drug permeability. Low permeability drugs are more likely to have poor absorption, distribution, metabolism, and excretion (ADME), which will reduce their effectiveness (17).

The process by which drugs cross the gastrointestinal barrier is intricate and involves a number of different mechanisms. To date, most drugs are absorbed by passive diffusion. In addition, different mechanisms, such as drug metabolism in the gastrointestinal system by enzymes and active transport via transporters, frequently occur simultaneously. These mechanisms can involve molecular interactions of various drug molecules with the gut's metabolizing enzymes, transporters, and physiological barriers of the gastrointestinal system. The physicochemical characteristics of the drug have a significant influence on these interactions (18). Therefore, one of the most important conclusions from pharmacokinetic research is that there is a strong association between the reported human effective permeability values and the degree of drug absorption.

## Methods for improving solubility and permeability

### Particle size reduction

One issue that frequently arises when creating drug delivery systems is that the active ingredient does not reach the target level required to elicit a therapeutic response. Various techniques can improve water solubility and oral bioavailability like micronization and particle size reduction. Decreasing the particle size of an active ingredient to approximately 2-5 µm is one of the oldest approaches to increase solubility (19). However, the solubility equilibrium of the drug cannot be increased using the micronization technique. In fact, by increasing the surface area/drug ratio, the dissolution rate can increase and the active ingredient can either dissolve or diffuse from the drug particles. Greater solubility is the outcome of more substantial interactions between the solvent molecules and a larger surface area. Thus, reducing the particle size can increase the solubility because of the increased surface area (20).

### Salt formation

The most popular technique for boosting the solubility and bioavailability of active substances in water is salt production. In order to improve the solubility of active medicinal components, the pharmaceutical industry has extensively employed salt formation techniques over the past 60 years. Currently, over half of the medications on the market are in salt form because they are easily formed during synthesis and crystallization. Moreover, the process of salt formation involves more than just changing the solubility and dissolving rates; it also involves a number of pharmacological and technological factors, including toxicity, physicochemical stability, impurity profile, and manufacturability (21).

**Figure 1:**
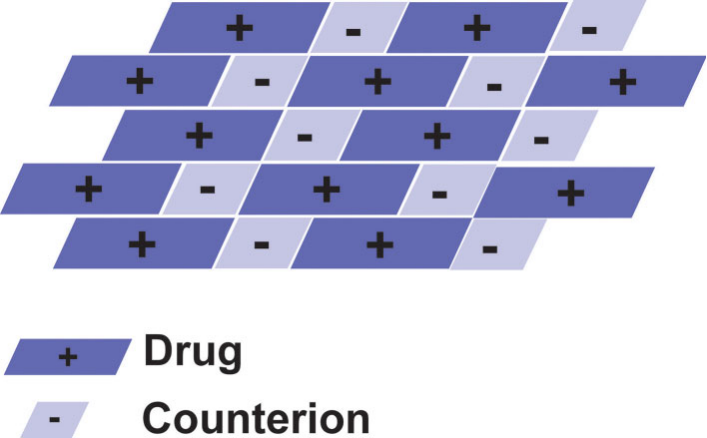
Schematic of the chemical structure of pharmaceutical salt formation.

Salt is a chemical compound consisting of a collection of cations and anions. Pharmaceutical salts are composed of counterions that can be either atomic (such as sodium bromide) or molecular (such as mesylate, acetate) and ionized active pharmaceutical compounds (anionic, cationic, or zwitterionic) (22). Sodium (Na^+^) and chloride (Cl^-^) are often utilized as counterions for pharmaceutical salts (23). Salt formation is schematically shown in Figure 1.

The salt formation method results in a high water solubility of the drug, which is suitable for increasing the solubility of therapeutic compounds. Formation of salts can increase dissolution rate compared to the solubility of the active ingredient. Salt solubility can be affected by the solubility of the active pharmaceutical ingredients and counterion salts (23).

### Nanoparticle drug carriers

Nanoparticle-based drug delivery systems are particulate or solid dispersions that are 10-1000 nm in size. The drug dissolves and gets trapped, encapsulated, or bonded to the matrix of nanoparticles (6). Using a variety of techniques, nanoformulation technology can decrease pharmaceuticals to nanosize for a number of benefits, including increased bioavailability and solubility, fewer systemic adverse effects, increased circulation duration, and precise prioritization of drug accumulation at the target site (24).

In this review, we discuss the application of drug delivery systems based on nanoparticles and nanocarriers to increase drug solubility, permeability, and bioavailability.

### Liposomes

Liposomes are vesicular drug delivery systems consisting of phospholipids forming an aqueous core in a bilayer membrane. Drugs that are hydrophilic or hydrophobic can become more soluble and permeable by using liposomes as a drug delivery method. This is due to the presence of a hydrophilic tail and a hydrophobic head of the phospholipid, which encapsulate the drug and form hydrogen bonds with water (25). Liposomes have greater qualities than traditional drug delivery methods, such as targeting systems, controlled or prolonged release, defense against drug clearance and degradation, increased therapeutic effectiveness, and less harmful side effects (26). A previous study found that formulating an antimalarial drug into liposomes can result in slower drug release (27,28). As mentioned by Miatmoko et al. (29), nanoparticulate carriers such as liposomes can be good systems for drug delivery in cancer therapy. The encapsulation of doxorubicin in liposomes using poly-glycolic acid (PGA) as the internal trapping agent leads to the accumulation and inhibition of tumor development. Drugs can be delivered passively or actively using liposome encapsulation, which reduces adverse effects and allows for drug delivery to the target site (30). The use of liposomes to enhance the bioavailability of drugs can be seen in Table 1.

**Table 1:** Use of liposomes in anticancer drug formulations to increase drug bioavailability.

Drug	Method	Formula	Characteristics	Result	Reference
6-shogaol	Thin film dispersion	- 6-shogaol (15 mg) - Phospholipid (120 mg) - TPGS (5 mg) - Cholesterol (20 mg) - Sodium cholate (105 mg) - Isopropyl myristate (63 mg)	6-shogaol liposome Particle size: 40.35 nm PDI: 0.242 Zeta potential: −37.47 mV EE: 92.32% DL: 4.29% 6-shogaol liposome coated TPGS Particle size: 23.50 nm PDI: 0.140 Zeta potential: −45.40 mV EE: 95.18% DL: 4.35%	The liposome formulation increased the solubility of 6-shogaol compared to its free form; it increased C_max_ 1.81-1.82 times, T_max_ 2 times, T_1/2_ 1.48-1.87 times, mean residence time (MRT) 1.48-1.82 times, and AUC_0-t_ 2.82-5.80 times compared with the respective values for 6-shogaol in the free form.	74
Azadiradione	Thin film hydration	- Lecithin (500 mg) - Cholesterol (125 mg) - Azadiradione (80 mg)	Particle size: 323.4±9.79 nm PDI: 0.594±0.0331 Zeta potential: −38.13±1.89 mV EE: 82.02±9.01 %	Compared to the oral free drug, the oral administration of liposomal formulation had a much better plasma bioavailability of azadiradione. It increased AUC 11.44-fold form and increase C max 1.6-fold compared to its free.	75
Catechin hydrate	Ethanol injection	- Phosphatidylcholine (0.5% w/v) - Cholesterol (25% w/w) - Stearic acid (10% w/w) - Phosphatidylserine (3% w/w) - Chitosan (0.1% w/v)	Particle size: 137.30 nm PDI: 0.15 Zeta potential: 36.80 mV EE: 61.83% DL: -	Chitosome increased drug permeability *ex vivo* in rat intestines by 6.2 times compared with free drug. The catechin hydrate formulation in the chitosome system also increased the bioavailability indicated by a 4 times increase in the T_max_, 2 times in the C_max_, and 2.12 times in the AUC_0-24_.	76

PDI: polydispersity index; EE: encapsulation efficiency; DL: drug loading.

### Niosomes

**Figure 2:**
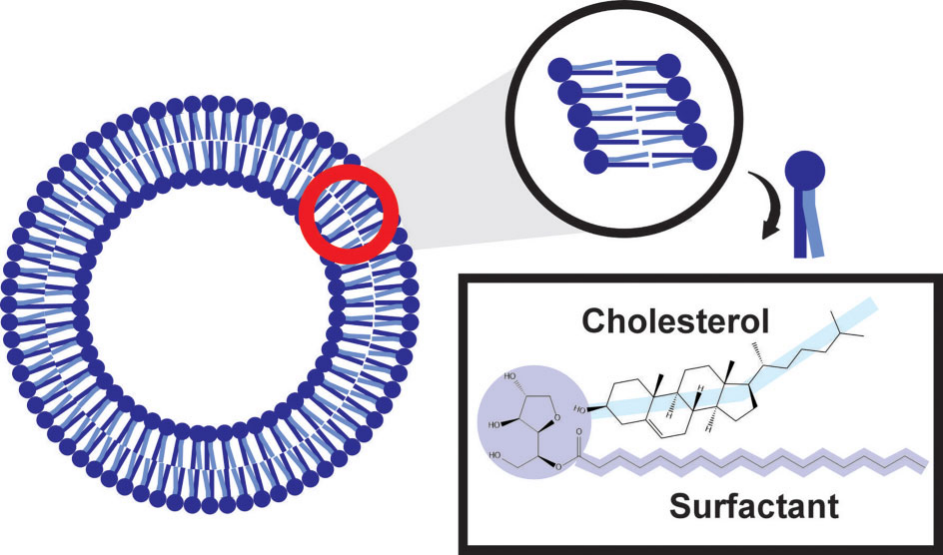
Schematic representation of niosome structure.

Niosomes are alternative carriers in drug delivery systems that have a physical structure identical to that of liposomes. This system is a dispersion of lipid vesicles with or without cholesterol, consisting of a non-ionic surfactant that is safe and biocompatible. Hydrophilic drugs may be added to the inner core of the lipid vesicles, while lipophilic drugs can be added to the exterior lipid bilayer (31). In addition, niosomes have a stable spherical bilayer structure consisting of nonionic surfactants and cholesterol (Figure 2). Niosomes are approximately 50-1000 nm in size and have amphitatic properties. Niosomes can encapsulate a variety of medications, improving the oral bioavailability of poorly absorbed pharmaceuticals because of their unique features that increase drug stability, oral bioavailability, and penetration (32), as presented in Table 2. In addition, the advantages of using niosomes as a drug delivery system compared to liposomes include lower production costs, better stability, better encapsulation, and easier production and storage. Drug delivery using niosomes can increase drug solubility because of their amphipathic nature. Water-soluble drugs are encapsulated in the core cavity of a niosome, whereas water-insoluble drugs are encapsulated in the nonpolar part of the bilayer (33).

**Table 2:** Use of niosomes in anticancer drug formulations to increase drug bioavailability.

Drug	Method	Formula	Characteristics	Result	Reference
Curcumin	Thin film hydration	- Curcumin - Cholesterol - Surfactants (span 20, span 60, span 80) - Dicetylphosphate - Lipid/drug mol ratio 10:1 - Surfactants: cholesterol: DCP molar ratio 2:1:0.05	Particle size: 218.30-333.20 nm PDI: 0.25-0.39 Zeta potential: 36.80 mV EE: ∼90% DL: -	Stability studies indicated that modification of niosomal formulations with PEG-FA is essential for minimizing problems associated with niosomal instability, such as aggregation, fusion, and drug leakage. Evaluation of the release showed that the niosome formulation produced a prolonged drug release. Effectiveness studies also showed increased anticancer properties through increased cytotoxicity, cell uptake, and cycle arrest activity.	77
Docetaxel	Thin film hydration	- Docetaxel - Cholesterol (2.5 mg) - Span 40 (2.5 mg)	Particle size: 244.9 nm PDI: 0.75 Zeta potential: −10 mV EE: ∼97.43 % DL: -	Docetaxel formulation in niosomes provides controlled release over a long period. Cytotoxicity evaluation showed that in MCF-7 cells, niosomes significantly enhanced cytotoxicity, showing sustained delivery of docetaxel over 24 h.	78
Ursolic acid	Thin film hydration	- Ursolic acid - Span 60 Cholesterol	Particle size: 255.1-439.3 nm with chitosan coating PDI: 0.298-0.565 Zeta potential: −46.23 to −20.89 mV EE: 11.8-16.0% DL: -	The addition of chitosan increases the physical characterization of niosome ursolic acid, affecting drug release from niosomes. *In vitro* study found that the addition of chitosan in niosomes increased drug concentration in the mice’s plasma and liver. In an effectivity study, ursolic acid niosomes with chitosan coating increased the effectiveness of preventive treatment in mice with NDEA-induced liver damage.	79-81

PDI: polydispersity index; EE: encapsulation efficiency; DL: drug loading.

Niosomes enhance the permeability of the drug in the gastrointestinal tract, as niosomes are lipophilic and have non-ionic surface-active substances. In addition to improving the gastrointestinal permeability, the lipophilic system of niosomes can improve the partitioning of the mucosal barrier, leading to improved permeability (34). Based on the research conducted by Attia et al. (35), the niosome formulation was found to be the cause of an increase in absorption triggered by a notable increase in C_max_ values. Span 60, a surfactant in niosome components, is believed to act as a penetration enhancer in the permeability of the gastrointestinal membrane. Furthermore, lymphatic transportation can be increased by using niosomes to avoid first-pass metabolism and increase bioavailability. Additionally, it was shown that Span 60 might induce ultrastructural alterations that temporarily open the tight junctions of the intestinal epithelial wall, hence enhancing the permeability of the intestinal epithelium (36).

### Polymeric micelles

Micelles are formed by surfactant molecules that are above the critical micelle concentration (CMC). Micelles are molecular aggregates with hydrophobic cores and hydrophilic surface (37). Hydrophobic compounds find a favorable environment in the hydrophobic interior of micelles, which can lead to a considerable increase in their solubility (37). For a long time, the primary drug delivery strategy for making hydrophobic drugs more soluble in water has been the use of surfactants. Surfactants are essential for giving poorly soluble drugs the solubilizing force they need to be absorbed orally (8).

The capacity of micelles to make insoluble materials more soluble is one of their key features. Solubilization is the spontaneous dissolution of a material in water by reversible interactions with surfactant micelles, resulting in the formation of an isotropic solution that is thermodynamically stable and has a lower thermodynamic activity. Micelles are spherical amphiphilic structures that have hydrophobic cores and hydrophilic shells. The hydrophobic core of the micelles carries a payload of therapeutic pharmaceuticals, while the hydrophilic coating makes the micelles water soluble (38).

Oral medication administration can be facilitated by polymeric micelles. By either enhancing the drug and carrier's membrane permeability or blocking the gastrointestinal mucosa's drug efflux transporters, this system can enhance drug absorption by the mucosa. In addition, the size of micelles favors their diffusion through the mucus layer. Thus, micelles increase drug solubility and permeability, increasing bioavailability.

The solubility of hydrophobic drugs can be considerably improved if they are formulated in a mixed micelle system (39). Ethenzamide and ibuprofen in combination micelles improved the solubility of the active components in water, just like in micellar drug delivery formulations (40). Previous studies have shown that surfactants can affect membrane permeability. They can increase the permeability of the gut membrane to drugs with a high water solubility and poor permeability (i.e., BCS class III compounds) by disrupting membrane integrity and enhancing paracellular transport, such as by opening tight junctions or inhibiting efflux transporters (8). The preclinical studies of polymeric micelles for delivering oral drugs can be seen in Table 3.

**Table 3:** Use of micelles in anticancer drug formulations to increase drug bioavailability.

Drug	Method	Formula	Characteristics	Result	Reference
Curcumin	Solvent evaporation	- Curcumin (10 mg) - Pluronic F-127 (150-250 mg) - Gelucire 44/14 (150-200 mg)	Particle size: 185-415 nm PDI: - Zeta potential: - EE: 35-76% DL: -	Formulation in the micelle system can provide increased drug permeation and diffusion. Curcumin in the micelle system exhibited a higher cytotoxicity effect due to increased drug concentration in the cells. The pharmacokinetic evaluation revealed a controlled profile with a three-fold rise in C_max_, 2-fold rise in T_max_, 1.33-fold rise in T_1/2_, and of 55.72 times rise in AUC compared with the respective values for free curcumin administered orally.	82
Paclitaxel	Ultrasonic emulsification	- Paclitaxel - Gallic acid - Chitosan - D-α-tocopherol polyethylene glycol 1000 succinate (TPGS) - Ratio drug:polymer 6:1	Particle size: 134.9 nm PDI: 0.172 Zeta potential: 34.8 mV EE: 80% DL: 8.2%	The paclitaxel formulation in a micelle system exhibited controlled drug release, which may be due to factors such as the hydrophobicity of the nucleus in the micelles and intermolecular hydrogen bonds. The micelle formulation also provided an increase in bioavailability shown by the increase of 3.95 times in C_max_, of 2 times in T_max_, of 1.56-fold in T_1/2_, and of 3.53 times in AUC_0-t_ compared with the respective values for free paclitaxel. This system also exhibited a higher antitumor effect due to increased absorption in the intestine and increased bioavailability, as well as because of the EPR effect.	83
Syringic acid	Thin film hydration	- Syringic acid (20 mg) - Pluronic F127 (240 mg) - Pluronic F68 (120 mg) - TPGS (120 mg)	Particle size: 21.18 nm PDI: 0.134 Zeta potential: −5.37 mV EE: 94.3% DL: 4.42%	Formulation of this drug in a micelle system resulted in increased cell uptake with increased drug accumulation in cells via the endocytosis pathway. Small micelle particle size increased the penetration ability resulting in higher drug accumulation. Pharmacokinetic studies showed that the use of micelles resulted in increased pharmacokinetic values, with 1.28 times increase in C_max_, 4.38 times increase in T_1/2_, 2.2 times increase in MRT, and 2.25 times increase in AUC_0-t_ compared with the respective values for the over-the-counter drug administered orally.	84
Aripriprazole	Thin film hydration	- Aripriprazole (5, 10, 15 mg) - Soluplus (300 mg) - TPGS (25 mg)	Particle size: 48.19-52.37 nm PDI: <0.3 Zeta potential: (−4.44) to (−4.75) mV EE: 98-99% DL: 1.5%	Drug formulation in the micelle system results in prolonged release of drugs, which indicates that the drug is more stable in the gastrointestinal tract, which can result in increased bioavailability. The permeability study showed that the micelle formulation increased by five times the drug's permeability in comparison to the over-the-counter medication. Pharmacokinetic studies also showed that the use of micelles increased the bioavailability, with 1.6 times increase in C_max_, 1.42 times increase in T_1/2_, and 1.6 times increase in AUC_0-24_ compared with the respective values for the free drug administered orally.	85

PDI: polydispersity index; EE: encapsulation efficiency; DL: drug loading.

### Complex inclusion

Another method for increasing solubility is the formulation of inclusion complexes using cyclodextrins (CDs). CDs are composed of a minimum of six d-(+)glucopyranose units connected by α-(1,4) linkages. Naturally occurring CDs with six, seven, eight, and nine glucose units are called α-, β-, γ-, and δ-CD (41). The truncated cone-shaped molecule HPβCD has a tapering hollow chamber that makes it possible to connect tiny molecules. Due to the presence of multiple hydroxyl groups, the surface of HPβCD is very hydrophilic, while its interior is modestly lipophilic. Because of this special characteristic, HPβCD is a good option for creating inclusion complexes with hydrophobic compounds. Furthermore, they have the ability to generate non-covalent and reversible inclusion complexes with substances that geometrically fit into their cavity (42).

**Figure 3:**
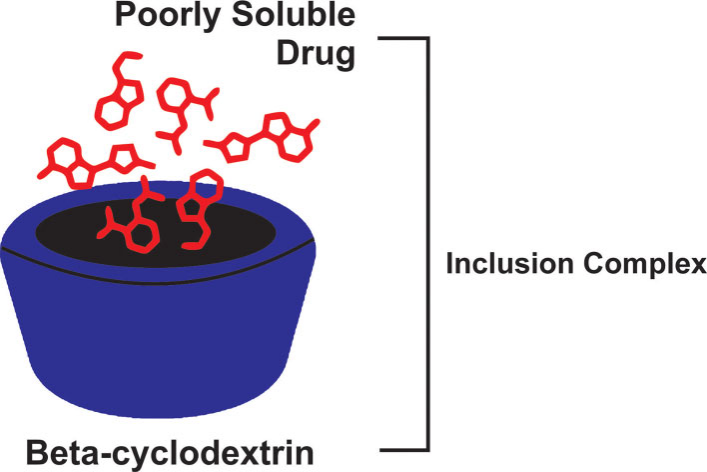
Mechanism underlying the increasing of the solubility of poorly soluble drugs by cyclodextrin through entrapment and complex formation with drug molecules.

Drug molecules can be trapped by CDs due to their hydrophobic core, as seen in Figure 3. CDs form inclusion complexes with drugs by encapsulating hydrophobic molecules inside the annulus. The formation of this combination improves the solubility of the medication and facilitates its passage through the undisturbed aqueous layer and onto the enterocytes' apical membrane. Furthermore, this inclusion complex raises the drug's permeability by enterocytes, improving the drug's pharmacokinetic profile and bioavailability (43).

Besides increasing drug solubility, the formulation of the CD inclusion complex is associated with the capability of cyclic oligosaccharides to promote membrane fluidization by removing phospholipids and membrane proteins from the apical membrane. Furthermore, the gastrointestinal tract's tight junctions may enlarge as a result of CD drug complexes, increasing paracellular transport (44). The formation of inclusion complexes also allows drugs to be easily transported in the gastrointestinal tract by increasing drug wettability, solubility, and dissolution rate, thus boosting the drug's stability and permeability through the gastrointestinal mucosa. Thus, drug formulation with CD inclusion complex system allows drugs with poor solubility and permeability to be delivered across lipophilic cell membranes, increasing bioavailability, as presented in Table 4.

**Table 4:** Use of inclusion complex in anticancer drug formulations to increase drug bioavailability.

Drug	Method	Formula	Characteristics	Result	Reference
Fisetin	Simple coacervation technique	- Fisetin (5 mg) - HPβCD (2-16 mM)	Particle size: 87.27 nm PDI: - Zeta potential: −8.71 mV EE: 79% DL: -	Formulation of this drug in an inclusion complex system increases the cytotoxicity of fisetin, which can be attributed to increased intracellular delivery via endocytic internalization into cells. Compared with the use of free fisetin, the inclusion complex formulation showed an increase of 8.8 times in C_max_, 1.32 times in T_max_, 1.80 times in T_1/2_, 1.67 times in MRT, and 15.67 times in AUC_0-t_.	42
Honokiol	Saturated aqueous solution	- HPβCD (2.89 g) - Honokiol (0.5 g)	-	The solubility of honokiol can be increased up to 1497 times through formulation in the inclusion complex.Pharmacokinetic studies showed that the formulation in the inclusion complex system increased the bioavailability by increasing C_max_ 7.22 times and AUC 4.78 times compared with the free drug administration. Moreover, compared to the free form, this system exhibited greater anticancer activity against the human hepatoma cell line (HepG2).	86
Pemetrexed	Coprecipitation method	- Pemetrexed (10 mg) - HPβCD (32.66 mg) - Polyoxypropylene glycol (P188) (10 mg)	Particle size: 14.5 nm PDI: 0.13 Zeta potential: −3.15 mV EE: >95% DL: -	The formulation in the inclusion complex system caused an increase of 29.5 times in membrane permeability compared with that for the free drug—this increase in permeability resulted in increased anticancer effectiveness and bioavailability. Pharmacokinetic studies showed an increase of 1.42 times in the bioavailability.	87

PDI: polydispersity index; EE: encapsulation efficiency; DL: drug loading.

### Solid lipid nanoparticles (SLN)

**Figure 4:**
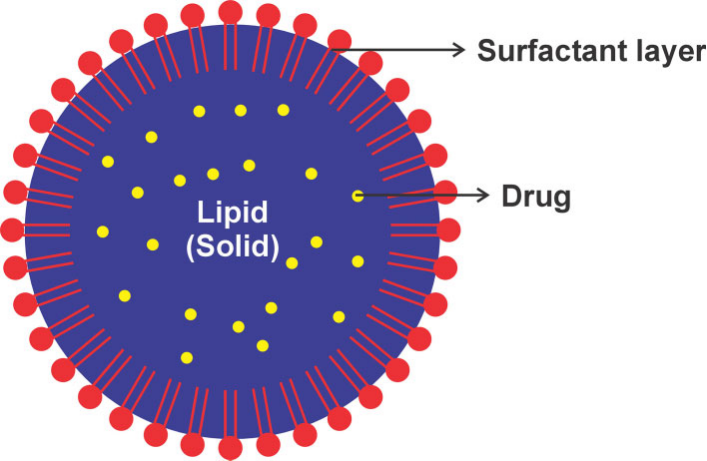
Schematic of the complete structure of solid lipid nanoparticles.

SLNs are colloidal systems derived from lipid matrices (Figure 4). These colloidal systems are made up of a lipid matrix that is distributed in an aqueous medium stabilized by surfactants and/or co-surfactants and maintains its solid state at room and body temperature. With the use of surfactants, this system may be dispersed in aqueous fluids with particle sizes ranging from 50 to 1000 nm (45). SLN formulations primarily consist of lipids in the solid state at room temperature, emulsifiers, active pharmaceutical ingredients (APIs), and suitable solvent systems (46). This technique was created to improve the absorption of drugs that are difficult to dissolve.

SLNs are an attractive drug delivery method, especially for drugs that are difficult to dissolve in water. Formulation of drugs with poor solubility using SLN can enhance the drug's rate of dissolution in the digestive system, thereby producing a concentration gradient that increases penetration into the gastrointestinal tract. The submicron size of SLN particles are responsible for the increased dissolution rate. The small particles have a large surface area that increases the drug dissolution rate (47). The use of SLNs for improving oral drugs' bioavailability is presented in Table 5.

**Table 5:** Use of solid lipid nanoparticles (SLNs) in anticancer drug formulations to increase drug bioavailability.

Drug	Method	Formula	Characteristics	Result	Reference
Abiraterone	Emulsion/ solvent evaporation	- Abiraterone acetate (10 mg)- Glyceryl monostearate (40 mg)- Soya lecithin (10 mg)- Tween 80 (1% w/w)- Poloxamer (1% w/w)	Particle Size: 197.2 nmPDI: 0.216Zeta Potential: 110 mVEE: 77%DL: -	Drug formulations in the SLN system exhibit higher levels of cytotoxicity because SLNs are more permeable and better absorbed and retained by cancer cells. Increased systems have also been reported using SLN permeation because their extremely lipophilic nature and nanometric size facilitate both transcellular and paracellular transit across intestinal epithelial cell layers and into the systemic circulation. Pharmacokinetic studies have shown an increase of 15.24 times in the C_max_ value and of 1.31 times in AUC_0-t_ compared with the respective values for the over-the-counter drug administered orally.	88
Cantharidin	Film dispersion- ultrasonication	- Cantharidin (2 mg)- Lecithin (4 mg)- Glyceryl monostearate (10 mg)- Cholesterol (4 mg)- Poloxamer 188 (0.5%)- Tween 80 (2%)	Particle size: 121 nmPDI: -Zeta potential: -23.09 mVEE: 93.83%DL: 13.28%	Formulations in the SLN system can increase drug solubility and release owing to their small size. Bioavailability studies showed that the SLN formulation increased drug absorption by 250.8% over that of the free oral form.	89

PDI: polydispersity index; EE: encapsulation efficiency; DL: drug loading.

## Potential uses of nanoparticles in maximizing drug bioavailability

The advantage of using nanotechnology lies in the physiochemical properties of nano-size materials and structures. Numerous aspects, including drug solubility, cellular absorption, membrane permeability, and biodistribution within the body, can be impacted by the size of nanoparticles. Therefore, the bioavailability of medications at the target location can be improved by using a nanoparticle drug delivery system. Herein, we explain the advantages of using nanoparticles in terms of their solubility, permeability, and stability.

### Nanoparticles enhance drug solubility (Figure 5)

Drugs usually formulated as nanoparticles have solubility problems that limit the amount of drug that is absorbed in the gastrointestinal system. All oral nanoparticle products are nanosuspensions, which are nanosized insoluble drug particles. Reducing drug particles to a nano size can increase their solubility. Smaller particles provide greater surface area and stronger contact with the solvent, which increases drug dissolution. Therefore, by increasing the surface area, more drug is absorbed via the normal absorption pathway. Because of the greater surface area, there might be more substantial interactions with the solvent, which increases solubility (48). Additionally, drugs that are difficult to dissolve with surfactants become more soluble when hydrophilic and hydrophobic groups are present. Because surface-active agents cause micelle formation, they increase the solubility of drugs that are not soluble in water. This process is referred to as micellar solubilization (49).

**Figure 5:**
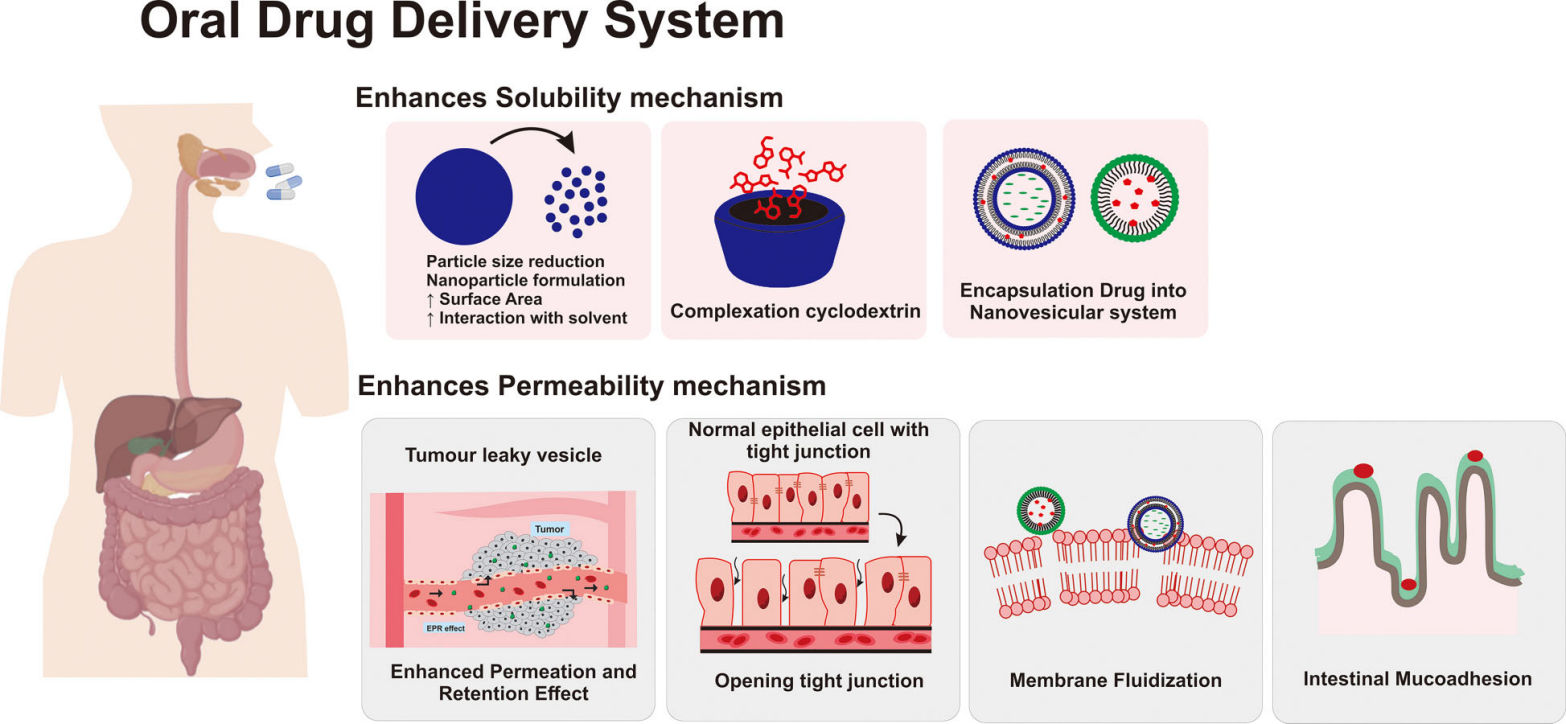
Mechanism underlying the enhancement of solubility and permeability of active pharmaceutical ingredients in nanoparticles.

Another possible mechanism involves the use of inclusion complexes. One way to create these systems is to insert a nonpolar molecule or a nonpolar portion of a molecule into the cavity of another molecule or group of molecules. The cavity of the host molecule must be both large enough to accommodate the guest molecule and small enough to drain water in order to reduce the overall amount of contact between water and the nonpolar areas of the host and guest. CDs are the most commonly used host molecules. CD complexation has been utilized to increase solubility (49).

Vesicular systems, such as liposomes and niosomes, are a novel way to deliver drugs in a controlled manner, improving bioavailability and achieving a longer therapeutic effect. Vesicular systems are lamellar structures with aqueous compartments surrounding amphiphilic molecules. Drugs that are both hydrophilic and hydrophobic and have been encapsulated in an outer lipid layer or inside a hydrophilic compartment can be delivered via vesicular systems. Because of their capacity to improve solubility, non-ionic surfactants are used to enhance the bioavailability of drugs that are water-insoluble (50).

### Nanoparticles enhance drug permeability (Figure 5)

Nanoparticles can enhance permeability in various ways. For example, the ability to load drugs into nanoscale-size particles helps protects anticancer drugs from degradation. Nanoparticles have a large surface area for conjugating targeted ligands, being able to release the drug in a controlled or prolonged way. Additionally, owing to limited lymphatic outflow in cancer cases, the nanosize is maintained in the tumor after selectively leaking via porous tumor capillaries and into the tumor tissue This phenomenon is known as the “enhanced permeability and retention (EPR) effect” (51).

Tight junctions between epithelial cells are a key component of the intestinal mucosal barrier. When intestinal cell tight junctions are compromised, permeability increases. The chitosan-shelled pH-responsive nanoparticle technology may successfully improve the drug's oral absorption by mediating tight junction openings (52).

Nanoparticles can also enhance permeability through permeability enhancers such as surfactants. The application of a surfactant that can fluidize membranes while altering the activity of efflux transporters increases the substrate's permeability across the membrane (53). Prior studies have linked the improvement in epithelial permeability caused by non-ionic surfactants to the formation of channels (pores) in the plasma membrane and the increased fluidity of cell membranes (54).

Nanocarriers with varying moieties and mucoadhesive characteristics can be functionalized to enable drug targeting, enhanced bioavailability, and biodistribution at the site of action, prolonging the drug's half-life for preferential accumulation, maximize therapeutic effect, and minimize systemic toxicity. Previous studies have reported that various kinds of nanoparticles and nanocarriers can increase drug bioavailability and enhance mucoadhesive properties (55). Mucoadhesive systems exploit the interactions between polymer-containing formulations and the mucus layer that coats the epithelial surfaces of the body. Mucoadhesive systems offer a viable method for enhancing drug absorption and boosting bioavailability owing to their longer retention times and close contact with absorption sites (56).

### Nanoparticles enhance drug stability

Nanoparticles also enhance drug stability. When employed as drug delivery vehicles, nanoparticles can prevent drug degradation and increase the efficacy of pharmacological treatments (57). It is challenging to administer drugs using nanoparticles orally because the gastrointestinal tract (GIT) is an obstacle, as the mucus barrier may shorten the time that oral nanoparticles remain in the body. However, nanoparticles that can penetrate mucus may enhance oral drug delivery. During oral delivery, nanoparticles may protect the drug from the harsh environment of the GIT and boost its bioavailability. By encapsulating medications in nanoparticles, pharmaceuticals can be made more soluble and bioavailable, they are better able to target particular GIT sites, and more stable in harsh GIT (58). Consequently, the medication can be protected along the GIT by using nanoparticles so that the active drug can remain high in the absorption site, and the drug can be absorbed faster than its conventional form.

## Challenges of nanoparticles for oral drug delivery system

Although nanoparticles have several benefits, they have various limitations, including complex manufacturing processes, nanotoxicity, and stability issues (59). Stability is crucial to ensure the effectiveness and safety of medicinal products, and stability issues can arise during the manufacturing, storage, and shipping processes of drug nanoparticles.

### Physical instability

Common physical instabilities of nanoparticle systems include sedimentation, agglomeration, aggregation, lysis, oxidation, and drug enzymatic exposure (59). Physical instability also occurs during dispersion, such as in liposomes and niosomes that tend to aggregate or fuse, causing leakage or hydrolysis of encapsulated drugs (60).

The enormous surface area of nanoparticles results in a high total surface energy, which is undesirable from a thermodynamic perspective. Nanoparticles are highly reactive due to the high number of weak bonds and surface imperfections. Due to their tiny grain size, nanoparticles have a high surface energy; hence, processes of nanoparticle assembly to lower surface energy can occur, in which particles aggregate to minimize surface energy. Agglomerates are loosely assembled groups of nanoparticles, whereas aggregates are firmly assembled groups of nanoparticles that are challenging to separate into individual particles with mechanical approaches (61).

Drug leakage is another type of instability. The leakage of water-soluble drugs from nanovesicles is a challenge for drug formulations, as it can lead to premature drug release, reduced effectiveness, and adverse effects (62). The common causes of nanovesicular drug leakage include membrane instability, inadequate encapsulation, and poor surface properties. The instability of the nanovesicle membrane can be caused by several factors including variations in temperature, pH, and mechanical stress. In addition, if a water-soluble drug is not adequately encapsulated within the nanovesicles, leakage may occur. Appropriate encapsulation methods are crucial for preventing drug leakage. Finally, the surface properties of nanovesicles may potentially affect drug leakage. For instance, the interaction between the nanovesicle surface and drug compounds may have an impact on the stability of the vesicle, leading to leakage. Therefore, understanding and addressing these factors is crucial for developing strategies to prevent drug leakage and improve the efficiency of nanovesicles for drugs delivery applications (62).

### Appropriate dosage form

Generally, nanoparticle drugs come in suspension form. Owing to its stability, this dosage form requires specific handling. For example, in the formulation of liposomes, the only option to overcome instability issues is to dry liposome-based formulations (63). However, the freeze-drying of nanoparticles can cause problems, such as colloidal instability, aggregation, and dispersion upon reconstitution. Freeze drying is a process aimed at removing the water from samples through sublimation and desorption under vacuum (64). To avoid long-term sedimentation problems, certain aqueous nanosuspensions are freeze- or spray-dried into a solid state. However, nonaqueous nanosuspension formulations cannot be prepared using the freeze-drying technique (59).

Various strategies, including formulation and process optimization, have been explored to overcome these challenges. Cryoprotective agents can be used to formulate nanoparticles before lyophilization. Adding sugar as a cryoprotectant is essential for maintaining the nanoparticle size and charge. The transition phase after rehydration of the nanoparticles can also be avoided by adding sugar. This is due to the fact that the sugar reduces the melting temperature upon drying. Disaccharides are favored over monosaccharides because of their higher glass-transition temperature (65).

### Production aspect

Nanoparticle production can be challenging for companies because nanodrugs are significantly more expensive than conventional medicines (66), because of the materials and methods used.

The manufacturing of nanoparticles differs from traditional manufacturing techniques in several ways. Nanoparticle production often requires multiple steps involving multicomponent systems, which can impact the process's repeatability and facilitate production scale-up (67). In addition, the manufacturing of nanoparticles requires advanced facilities and equipment. Nanomanufacturing processes face sterilization issues and regulatory requirements such as those in the Good Manufacturing Practices regulations. Furthermore, the production of nanoparticles requires much higher manufacturing accuracy, with the main challenge being the need for robust and reproducible processes (68,69).

These are some of the most important factors in the production of drug delivery systems based on nanoparticles and nanovesicles. Overall, the challenges in the production of nanoparticles underscore the need for innovative and efficient production methods to fully exploit the potential of nanomaterials.

### Clinical aspects

Nanoparticles have a high tendency to be absorbed in tissues, which modifies the pharmacodynamic and pharmacokinetic properties of formulated conventional/traditional drugs. When developing a drug delivery system, it is important to consider that the drug's therapeutic efficacy depends on its ability to be delivered selectively to a certain part of the body. The capacity of a medication to drive cancer cells into apoptosis determines the efficacy of cancer treatment. In addition, minimal side effects are significant in this case. High drug dosages increase the toxicity to healthy cells and raise the risk of drug resistance. Thus, the use of nanoparticles at lower doses can overcome this challenge while avoiding toxicity to normal cells (70).

The optimal dose of nanoparticles for specific applications depends on several factors such as surface characteristics, size and form of the nanoparticles, and the target tissue or organ (71). Changing the dosage of nanoparticles can affect their toxicity. Because nanoparticle toxicity is frequently dose-dependent, the amount of nanoparticles administered can affect the particles' toxicity. For instance, it was stated that cytotoxicity depends on the particle size and dose, with larger particles being more toxic than smaller particles at lower doses (72).

Thus, it is essential to thoroughly evaluate the potential toxicity and effectiveness of nanoparticles at various doses and carefully consider the dose of nanoparticles used in any application. Understanding the connection between nanoparticle dose and response and creating safe products for various applications are the primary objectives of continuing research.

## Nanoparticles in the current clinical setting

The versatility of nanoparticles in terms of form, charge, stability, and specific binding ability led to the development of novel therapeutic medicines and a more practical approach to cancer treatment. With several benefits that increase therapeutic effectiveness while reducing adverse effects, nanoparticles have emerged as a significant advance in anticancer therapy. An improved cancer treatment and an efficient substitute for chemotherapy are being developed through the use of nanotechnology. Because of their large surface area, nanoparticles can be functionalized with various ligands such as antibodies, peptides, or small molecules. This characteristic improves the pharmacokinetic characteristics and effectiveness of substances with active theranostic capabilities in the treatment of cancer.

Recent studies have shown that many nanoformulations are already significant in cancer treatment. Currently, a wide variety of drugs based on liposomes are accessible for human use. The majority of liposomal medication formulations are suitable for intramuscular and intravenous administration (73). However, the lack of Food and Drug Administration (FDA)-approved drugs or clinical studies for oral nanoparticles used in cancer treatment highlights the existing gaps in cancer treatment.
